# Extended oral antibiotics fail to reduce surgical site infection in orthopedic surgeries: A comparative study

**DOI:** 10.1371/journal.pone.0330685

**Published:** 2025-09-05

**Authors:** Alireza Mirahmadi, Pooya Hosseini-Monfared, Mehrdad Farrokhi, Raza Minaie, Amin Bateni, Seyed Morteza Kazemi

**Affiliations:** 1 Bone Joint and Related Tissues Research Center, Shahid Beheshti University of Medical Sciences, Tehran, Iran; 2 Student Research Committee, Department of Epidemiology, School of Public Health and Safety, Shahid Beheshti University of Medical Sciences, Tehran, Iran; IRCCS Istituto Ortopedico Rizzoli, ITALY

## Abstract

**Background:**

Surgical site infection (SSI) is associated with a significant burden in orthopedic surgeries, leading to increased morbidity, prolonged hospital stays, and higher healthcare costs. Despite the widespread use of prophylactic antibiotics to reduce the risk of infection, the optimal duration for antibiotic administration remains controversial. Newer studies reported controversial results compared to existing guidelines; therefore, we aimed to compare the efficacy and post-operative complications of short-term (<24 hours) and extended oral antibiotics in reducing infection rates following orthopedic surgeries.

**Methods:**

In this retrospective study, patients who underwent orthopedic surgeries, including total knee arthroplasty (TKA), total hip arthroplasty (THA), anterior cruciate ligament (ACL) reconstruction, and hip internal fixation, were recruited from the hospital data registry. Patients were divided into two groups of short-term (<24 hours) and extended oral antibiotics groups based on the duration of prophylactic antibiotic use. The infection rate during three months after the surgery and the incidence of antibiotic-related adverse events were compared between the groups.

**Results:**

Of the 398 patients in the study, 246 received short-term, and 152 received extended oral antibiotics. There was no significant difference between the short-term (2.8%) and extended (4.6%) groups with respect to the rate of SSI (P = 0.35). The patients in the extended antibiotic group demonstrated more post-operative complications compared to the short-term group (36.2% Vs. 22.8%, P = 0.004).

**Conclusion:**

Our findings demonstrated that extended oral antibiotics did not reduce the rate of SSI following orthopedic surgeries compared to short-term prophylaxis. Furthermore, patients who received prolonged antibiotic demonstrated a higher incidence of postoperative complications. Our study supports the recommendation not to use extended prophylactic oral antibiotics over 24 hours in orthopedic surgeries.

## Introduction

Surgical site infections (SSI) are associated with high health and economic burden, accounting for 40% of all healthcare-associated infections in surgery wards [1,2]. The prevention of SSI is of great importance as they are associated with reduced health-related quality of life, physical function limitation, and higher economic burden [3–5]. Preoperative antimicrobial prophylaxis has been introduced to reduce the rate of SSI following surgeries and has been shown to be effective [6]. Recent guidelines recommend first or second-generation cephalosporins as the optimal choice of prophylactic antibiotics [7]. First and second-generation cephalosporins are broad-spectrum antibiotics that primarily target aerobic Gram-positive and Gram-negative bacteria [8]. They exhibit excellent bactericidal activity, distribute well in bony, synovial, and muscle tissues, have low systemic toxicity, and are reasonably priced. First and second-generation cephalosporins exhibit a half-life that covers the critical time interval for SSI, which is typically two hours after incision or contamination [9]. Cefazolin has been the most extensively investigated agent in clinical trials [9–11]. The purpose of antibiotic prophylaxis such as cefazolin is to have proper minimum serum and tissue concentration levels for encountering microorganisms both during and after surgery [12,13].

To achieve the goal of having high antibiotic concentration levels in tissues at the time of incision, it is recommended that prophylactic antibiotics be administered within 30 minutes of skin incision [14]. The 2017 Centers for Disease Control and Prevention (CDC) Guideline for preventing SSI recommends not using further antibiotics after the surgical incision is closed in clean and clean-contaminated surgical procedures [15]. However, it should be mentioned that the CDC recommendations are mainly based on studies of trunk surgeries without any hardware retention, such as general surgery, cardiothoracic, and ear, nose, and throat surgeries. For orthopaedic surgery of extremities with hardware and implant retention, limited studies with insufficient power have evaluated the effect of antibiotic duration on SSI [5,16,17]. Furthermore, there have been reports of reduced infection rates following total joint arthroplasty in high-risk patients with extended oral antibiotics, which are against the recommendations of currently accepted guidelines [18–20].

Regarding the duration of prophylaxis, extended prophylactic antibiotic therapy (>24 hours) has been suggested for high-risk patients undergoing total joint arthroplasties [18,21,22]. However, the optimal duration of prophylactic intravenous antibiotics remains controversial, with some studies opposing extended prophylaxis [23]. Therefore, we aim to compare the impact of extended oral antibiotics compared to short-term cefazolin prophylaxis on the complications associated with orthopedic surgeries. We hypothesized that using extended antibiotics would not significantly affect SSI incidence.

## Methods

### Study characteristics

In this retrospective cohort study, we evaluated 398 patients who underwent orthopedic surgeries such as total knee arthroplasty (TKA), total hip arthroplasty (THA), anterior cruciate ligament (ACL) reconstruction, and hip internal fixation (HIF) at our hospital from January 2023 to January 2024. Inclusion criteria consisted of patients who had primary case orthopedic surgery; exclusion criteria were patients who underwent revision surgery, surgeries involving open fractures, patients with an active infection at the time of surgery, those receiving antibiotics for other indications, allergies to antibiotics, patients in pregnancy or lactation period, and patients with incomplete or missing medical records. The study was conducted per the ethical guidelines outlined in the Declaration of Helsinki ensuring that participant confidentiality and data protection were maintained throughout the research process. This study was approved by the ethics committee of Shahid Beheshti University of Medical Sciences (IR.SBMU.RETECH.REC.1397.1009). Consent for publication was obtained from all patients whose data were included in this study. The need for informed consent was waived due to the study’s retrospective nature. Data from medical records were accessed for research purposes in February 2024. The authors did not have access to any information that could identify individual participants during or after data collection, ensuring the confidentiality and anonymity of all patient data.

### Grouping

The patients were categorized into two groups according to the duration of antibiotic prophylaxis documented in hospital medical records as short-term (≤ 24 hours) and extended oral antibiotic (> 24 hours) groups. Patients in the short-term group received 1g of cefazolin 30 minutes before the surgery and further doses of cefazolin 6 and 12 hours after the surgery. For patients in the extended oral antibiotic group, the antibiotics were continued using oral cephalexin for up to 7 days. The postoperative infection rate was evaluated in two groups with different durations of antibiotic prophylaxis, and patients were evaluated for complications using electronic hospital records and telephone follow-ups. Data collection was conducted utilizing a researcher-designed questionnaire.

### Outcome measures

The primary outcome measure of this study was the infection rate after the surgery. Patients were evaluated for infection-related signs such as fever, pain, redness, warmness, itching, swelling, and infectious discharge were evaluated in the postoperative period. Furthermore, the length of hospital stay and postoperative antibiotic-related complications, including skin allergies, headache, vertigo, nausea, vomiting, diarrhea, and abdominal pain, were recorded. The patients were followed up to 3 months after surgery.

### Data analysis

Statistical analysis was performed using SPSS statistical software version 29. The Kolmogorov-Smirnov test was used to check the normality of the variables. Students’ t-test was used to analyze normally distributed continuous variables. In addition, we used the Mann-Whitney U test to analyze non-normally Distributed Continuous Variables. The Pearson Chi-Square test was used for categorical variables analysis, and Spearman and Pearson’s correlation tests assessed associations between the variables. P values less than 0.05 were considered statistically significant.

## Results

Finally, 398 patients who underwent orthopedic surgeries such as TKA (n = 113), THA (n = 61), Hip internal fixation (n = 118), and ACL reconstruction (n = 106) were assessed in the short-term (n = 246) and extended oral antibiotics (n = 152) groups. There was no significant difference between short-term and extended groups with respect to the mean age of the patients (54.5 ± 21.0 vs 58.1 ± 20.7, P-value = 0.129). Additionally, in the short-term group, 139 patients (56.5%) were male, as opposed to 73 (48.0%) in the extended group, showing no significant difference (P-value = 1). Other demographic characteristics of the included patients are summarized in Table 1.

**Table 1:** Basic characteristics of the included patients.

Characteristics	Short Term Group	Extended Group	P-value
Age (years)	54.52 ± 21.03	58.16 ± 20.77	0.129
Gender (M/F)	139/107	73/79	1
Length of Hospitalization (days)	5.48 ± 2.30	6.88 ± 2.70	<0.001
**Type of Surgery**			
TKA	62 (25.2%)	51 (33.6%)	0.005
THA	32 (13.0%)	29 (19.1%)	
ACL Reconstruction	80 (32.5%)	26 (17.1%)	
Hip Internal Fixation	72 (29.3%)	46 (30.3%)	

Notes: M/F: Male/Female; TKA: Total knee arthroplasty; THA: Total hip arthroplasty; ACL: Anterior cruciate ligament; PMH: Past medical history.

Since the proportion of the patients in the two groups differed in each surgery (Table 1), the results were analyzed and reported separately. The patient-related risk factors for SSI have been summarized in (Supplementary 1 in S1 File). Age, BMI, smoking status, comorbidities like DM, cardiovascular disease, and history of immunosuppressive drugs were similar between the extended oral antibiotics and short-term groups (P-value > 0.05) (Supplementary 1 in S1 File).

However, surgery-related factors, such as the length of hospitalization in patients who underwent TKA and ACL reconstruction, were significantly shorter in the short-term group than in the extended group (P-value < 0.05). Also, the duration of surgery in patients who underwent TKA was significantly shorter in the short-term group compared to the extended group (P-value = 0.014).

The results of SSI in two groups are shown according to the type of surgery in Table 2. The rate of SSI was not statistically different between the extended (4.6%) and short-term (2.8%) groups in the surgeries evaluated in our study (P-value = 0.35)(Table 2).

**Table 2:** The surgical site infection (SSI) results in two groups are shown according to the type of surgery.

Variables	TKA		p-value	THA		p-value	ACL Reconstruction		p-value	HIF		p-value
	Short	Extended		Short	Extended		Short	Extended		Short	Extended	
**SSI**	5 (8.1%)	4 (7.8%)	1	1 (3.1%)	1 (3.4%)	1	1 (1.3%)	1 (3.8%)	0.432	0	1 (2.2%)	0.390
**Complications**												
DVT	1 (1.6)	2 (3.9)	0.588	0 (0)	0 (0)	–	0 (0)	0 (0)	–	0 (0)	0 (0)	–
PTE	0 (0)	1 (2.0)	0.451	0 (0)	0 (0)	–	0 (0)	0 (0)	–	0 (0)	0 (0)	–
Hematoma	2 (3.2)	0 (0)	0.500	0 (0)	1 (3.4)	0.475	0 (0)	1 (3.8)	0.245	0 (0)	0 (0)	–
Pain	0 (0)	0 (0)	–	0 (0)	1 (3.4)	0.475	0 (0)	0 (0)	–	0 (0)	0 (0)	–

SSI: Surgical site infection; TKA: Total knee arthroplasty; THA: Total hip arthroplasty; ACL: Anterior cruciate ligament; HIF: Hip internal fixation, PTE: pulmonary thromboembolism, DVT: Deep vein thrombosis.

The overall rate of symptoms of post-operative complications was significantly higher in the extended group (36.2%) compared to the short-term group (22.8%) (P-value = 0.004, OR=1.9, 95%CI = 1.2–3.0). The swelling was significantly more prevalent in the extended group than the short-term group (P-value=0.022). We also analyzed each adverse effect related to the use of prophylactic antibiotics, such as headache, constipation, nausea, vomiting, diarrhea, loss of appetite, abdominal pain, and skin allergy in each surgery, and the results demonstrated no significant differences between the short-term and extended oral antibiotics groups in separate surgeries (P-value > 0.05) (Supplementary 2 in S1 File).

We also recorded the readmission causes after the surgery, and our findings demonstrate that the extended oral antibiotics group and the short-term group did not have any significant difference in the incidence rate of deep vein thrombosis (DVT), pulmonary thromboembolism (PTE), hematoma, and the pain that led to readmission (P > 0.05) (Table 2).

## Discussion

Prophylactic antibiotics are widely used to prevent SSI after orthopaedic surgeries, but the optimal duration of prophylactic antibiotic therapy remains a topic of debate. Our findings revealed that the rate of SSI was not significantly different between patients who received short-term (<24h) or extended prophylactic antibiotics after the orthopedic surgeries. However, the overall rate of post-operative complications, such as swelling, was significantly higher in the extended group than in the short-term group.

The duration of prophylactic antibiotic administration has been evaluated in fracture surgeries. A study comparing the efficacy of cefuroxime over 24 hours with the regimen of cefuroxime for 24 hours plus 6 days of oral cephalexin for patients with trochanteric fractures demonstrated that there were no differences between the two groups considering the infection rate [24]. A subsequent meta-analysis demonstrated that the multiple-dose antibiotic prophylaxis in closed long bone fractures was not superior to the single-dose strategy [25]. In this study, we separately compared the short-term and extended oral prophylactic antibiotics in patients who underwent hip internal fixation and found no significant difference between these two groups regarding infection rate.

Also, there is a discrepancy regarding the effect of extended antibiotics for arthroplasty surgeries. A retrospective cohort study of total knee and hip arthroplasties comparing a single preoperative dose of cefazolin and 3 doses of cefuroxime found that the rate of both superficial and deep wound infection was around 1% and not significantly different between the two groups [26]. A retrospective study by Inabathula et al. in high-risk patients for periprosthetic joint infection (PJI) demonstrated that extended oral antibiotics for seven days after discharge can reduce the infection rate following total hip and knee arthroplasties [18]. Similarly, a study by Kheir et al. in 2022 also demonstrated that extended oral antibiotics for 7 days could reduce the rate of PJI in high-risk patients from 2.64% to 0.89% [19]. They found that high-risk patients were nearly 4–5 times more likely to develop PJI after total knee or hip arthroplasties. Optimization of some of these factors can reduce the risk of postoperative infection, but not all patient-related risk factors can be modified [27]. Therefore, there may be a ceiling effect on the ability to reduce the infection rate by modifying the patient-related risk factors, and there might be an appropriate indication for extended oral antibiotics for this special population.

Based on previous studies, a review by Tucci et al. [28] reported that in orthopedic surgeries, short-term cefazolin prophylaxis’s efficacy is similar to extended prophylaxis to prevent SSI. They mentioned that due to the increased duration and costs of hospitalization and the risk of systematic toxicity, prolonging antibiotic prophylaxis beyond the first day is not necessary for preventing SSI. They also mentioned that prolonging antibiotic prophylaxis could increase colitis by *C. difficile*, negatively affecting individual and community microflora and, finally, facilitate the rise of pharmacological resistance. Moreover, similar to our findings, a study based on the Norwegian Arthroplasty Registry demonstrated that extended antibiotic prophylaxis beyond one day after surgery does not affect the SSI rate [29]. They also recommended first- or second-generation cephalosporins to prevent SSI [29,30].

A recent study by Chandrak et al. also demonstrated that extended oral antibiotics were not associated with a significant reduction in SSI in both implant and non-implant groups of orthopaedic surgeries [31]. Furthermore, they observed higher complications in the extended antibiotic group, including gastrointestinal upset and allergic reactions [31]. In our study, extended oral antibiotics showed a similar lack of benefit in reducing SSI rates compared to short-term antibiotics, although the incidence of gastrointestinal and allergic adverse effects was comparable between groups.

Antimicrobial resistance is one of the most important concerns about the extended use of prophylactic antibiotics. In addition to concerns about antimicrobial resistance, the possibility of adverse drug events must be considered. One of the main side effects of prolonged antibiotic therapy is reported to be *Clostridium difficile*-associated diarrhea [32]. Our findings demonstrated that the overall rate of complications was significantly higher in the extended group (35.5%) compared to the short-term group (22.8%). The swelling was significantly more prevalent in the extended group than the short-term group. The increased prevalence of swelling may explain the prolonged use of antibiotics to manage potential postoperative complications. Also, patients in the extended oral antibiotic group had longer hospital stays compared to the short-term group. Our findings demonstrated no significant difference regarding diarrhea between the short-term and extended groups.

Recent studies have shown that the prescription rate of extended oral antibiotics for primary THA increased by 366% from 2010 to 2022, and currently, 16.5% of patients undergoing outpatient THA and TKA are prescribed an oral antibiotic [33,34]. Certainly, it is imperative to note that, as per this study’s outcomes, prolonged antibiotic administration did not yield clinical benefits antibiotics and may not be fully effective in eliminating all SSIs. Consequently, caution should be exercised regarding the escalating utilization of antibiotics over extended periods. Given that most studies are retrospective, it is crucial to take into account the rationale behind surgeons prescribing extended antibiotics. These reasons should be factored in when drawing conclusions about the effectiveness of prolonged antibiotic use.

Additionally, the findings of this study revealed that despite the absence of a significant difference in SSI rates between the two groups, the extended antibiotic group had a higher infection rate (4.6% vs. 2.7%). This underscores the importance of exploring additional therapeutic interventions alongside, rather than solely relying on, prolonged antibiotic regimens. It should be noted that not all SSIs can be prevented with current evidence-based strategies, and only about 55% of them can be prevented with different antibiotic prophylactic strategies [35]. Optimizing perioperative antibiotics for orthopedic surgeries is a multifaceted process that does not have a universal solution. The details involved in decision-making related to antibiotic administration require explicit yet adaptable guidelines to accommodate the diverse characteristics of orthopedic patients [36].

Our study had some limitations as the study design was retrospective, and the related bias of the study design applies to this study. Future large-scale, multicenter, and randomized controlled trials are needed to provide high-quality evidence to support our findings. Secondly, the data on the indications for extended prophylactic antibiotics were not available. Therefore, addressing the underlying reason that the surgeon gave extended antibiotics to some patients may significantly affect the result and conclusion. Moreover, our sample size was heterogeneous as we included patients who were candidates for different types of orthopedic surgeries. It should be noted that while we reported differences in post-operative complications between the short-term and extended oral antibiotic group, these findings should be interpreted as observational associations rather than evidence of causation. We suggest that future studies assess the effect of the duration of prophylaxis on the rate of SSI in homogenous patients concerning the type of orthopedic surgery. Also, further studies are needed to evaluate the long-term impact of perioperative antibiotic duration on antimicrobial resistance patterns.

## Conclusion

We compared the postoperative infection rate and complications in two groups of patients who received short-term or extended oral antibiotics following four orthopedic surgeries. Our results indicated prolonged prophylactic antibiotics are ineffective in reducing surgical site infections following these orthopedic surgeries. Furthermore, the patients who received extended oral antibiotics presented with more postoperative complications compared to the short-term antibiotic group. Based on the findings of our study, extended oral antibiotics beyond 24 hours after the surgery are not recommended.

## Supporting information

S1 File Patient-related risk factors for SSI and Postoperative complications. (DOCX)
