# Supplementary material for: Extended oral antibiotics fail to reduce surgical site infection in orthopedic surgeries: A comparative study
Source: PLoS One. 2025 Sep 5;20(9):e0330685. doi: 10.1371/journal.pone.0330685 (PMC12412922; doi:10.1371/journal.pone.0330685)
Supplement: S1 File — (DOCX) [file pone.0330685.s001.docx]

Supporting Information

**S1. Patient-related risk factors for SSI**

| **Variables** | **TKA (113)** | | | **THA (61)** | | | **ACL (106)** | | | **HIF (118)** | | |
| --- | --- | --- | --- | --- | --- | --- | --- | --- | --- | --- | --- | --- |
| **Groups** | **Short**  **(62)** | **Extended**  **(51)** | **P Value** | **Short**  **(32)** | **Extended**  **(29)** | **P Value** | **Short**  **(80)** | **Extended**  **(26)** | **P Value** | **Short**  **(72)** | **Extended**  **(46)** | **P Value** |
| **Gender**  Male  Female | 11 (17.7)  51 (82.3) | 4 (7.8)  47 (92.2) | 0.123 | 20 (62.5)  12 (37.5) | 17 (58.6)  12 (41.4) | 0.757 | 77 (96.3)  3 (3.8) | 26 (100)  0 (0) | 1 | 31 (43.1)  41 (56.9) | 26 (56.5)  20 (43.5) | 0.153 |
| **Age (year)** | 66.95±6.25 | 66.57±6.94 | 0.758 | 55.31±16.87 | 53.66±20.39 | 0.730 | 29.58±5.47 | 30.15±11.65 | 0.719 | 71.18±14.77 | 67.52±20.59 | 0.740 |
| **BMI** | 27.77±3.83 | 28.97±4.20 | 0.125 | 24.46±3.62 | 23.89±2.66 | 0.533 | 25.84±3.22 | 25.88±2.77 | 0.620 | 25.11±3.75 | 25.35±4.17 | 0.676 |
| **Smoker** | 3 (4.8) | 1 (2.0) | 0.626 | 6 (18.8) | 7 (24.1) | 0.608 | 15 (18.8) | 3 (11.5) | 0.552 | 9 (12.5) | 11 (23.9) | 0.107 |
| **Alcohol Users** | 2 (3.2) | 0 (0) | 0.500 | 0 (0) | 1 (3.4) | 0.475 | 4 (5.0) | 1 (3.8) | 1 | 5 (6.9) | 1 (2.2) | 0.402 |
| **Comorbidities** |  |  |  |  |  |  |  |  |  |  |  |  |
| Diabetes (DM) | 9 (14.5) | 10 (19.6) | 0.471 | 3 (9.4) | 8 (27.6) | 0.065 | 0 (0) | 0 (0) | 1 | 20 (27.8) | 11 (23.9) | 0.642 |
| RA | 1 (1.6) | 3 (5.9) | 0.326 | 1 (3.1) | 4 (13.8) | 0.182 | 0 (0) | 0 (0) | 1 | 3 (4.2) | 2 (4.3) | 1 |
| **Immunosuppressive Drug Users** | 3 (4.8) | 2 (3.9) | 1 | 3 (9.4) | 5 (17.2) | 0.460 | 0 (0) | 0 (0) | 1 | 3 (4.2) | 2 (4.3) | 1 |
| **Anesthesia**  Spinal  General  Mix | 52 (83.9)  5 (8.1)  5 (8.1) | 43 (84.3)  3 (5.9)  5 (9.8) | 0.865 | 23 (71.9)  7 (21.9)  2 (6.3) | 22 (75.9)  5 (17.2)  2 (6.9) | 0.904 | 69 (86.3)  6 (7.5)  5 (6.3) | 18 (69.2)  7 (26.9)  1 (3.8) | **0.036*** | 63 (87.5)  5 (6.9)  4 (5.6) | 44 (95.7)  1 (2.2)  1 (2.2) | 0.425 |
| **Surgery Duration (min)** | 177.1±36.8 | 194.2±46.4 | **0.014*** | 159.3±47.8 | 174.6±34.9 | 0.116 | 123.5±33.6 | 135.9±28.0 | 0.054 | 136.3±43.6 | 140.1±39.0 | 0.521 |
| **Intraoperative Transfusion** | 4 (6.5) | 7 (13.7) | 0.219 | 7 (21.9) | 17 (58.6) | **0.003*** | 1 (1.3) | 0 (0) | 1 | 20 (27.8) | 17 (37.0) | 0.295 |
| **Length of Hospitalization (days)** | 5.89±1.70 | 7.22±2.20 | **0.002*** | 7.53±1.70 | 8.34±3.59 | 0.971 | 3.35±1.19 | 4.00±0.98 | **0.003*** | 6.57±2.15 | 7.20±2.01 | 0.132 |

**S2 – Postoperative Adverse Effects**

| **Variables** | **TKA (113)** | | | **THA (61)** | | | **ACL (106)** | | | **HIF (118)** | | |
| --- | --- | --- | --- | --- | --- | --- | --- | --- | --- | --- | --- | --- |
| **Antibiotic Duration** | **Short (62)** | **Long (51)** | **P Value** | **Short (32)** | **Long (29)** | **P Value** | **Short (80)** | **Long (26)** | **P Value** | **Short (72)** | **Long (46)** | **P Value** |
| Nausea | 7 (11.3) | 5 (9.8) | 0.799 | 2 (6.3) | 6 (20.7) | 0.135 | 1 (1.3) | 1 (3.8) | 0.432 | 7 (9.7) | 6 (13.0) | 0.574 |
| Vomiting | 2 (3.2) | 3 (5.9) | 0.656 | 1 (3.1) | 4 (13.8) | 0.182 | 1 (1.3) | 1 (3.8) | 0.432 | 5 (6.9) | 1 (2.2) | 0.402 |
| Diarrhea | 0 (0) | 0 (0) | - | 0 (0) | 0 (0) | - | 1 (1.3) | 0 (0) | 1 | 0 (0) | 0 (0) | - |
| Constipation | 3 (4.8) | 3 (5.9) | 1 | 1 (3.1) | 0 (0) | 1 | 0 (0) | 0 (0) | - | 2 (2.8) | 3 (6.5) | 0.377 |
| Abdominal Pain | 0 (0) | 1 (2.0) | 0.451 | 0 (0) | 1 (3.4) | 0.475 | 1 (1.3) | 0 (0) | 1 | 0 (0) | 1 (2.2) | 0.390 |
| L Appetite | 1 (1.6) | 1 (2.0) | 1 | 0 (0) | 0 (0) | - | 0 (0) | 0 (0) | - | 0 (0) | 1 (2.2) | 0.390 |
| Weakness (LOC) | 1 (1.6) | 4 (7.8) | 0.173 | 1 (3.1) | 0 (0) | 1 | 0 (0) | 1 (3.8) | 0.245 | 2 (2.8) | 2 (4.3) | 0.642 |
| Tachycardia | 3 (4.8) | 4 (7.8) | 0.699 | 2 (6.3) | 0 (0) | 0.493 | 1 (1.3) | 0 (0) | 1 | 1 (1.4) | 3 (6.5) | 0.298 |
| Tachypnea | 0 (0) | 1 (2.0) | 0.451 | 0 (0) | 1 (3.4) | 0.475 | 0 (0) | 0 (0) | - | 2 (2.8) | 2 (4.3) | 0.642 |
| Dyspnea | 3 (4.8) | 5 (9.8) | 0.464 | 1 (3.1) | 0 (0) | 1 | 2 (2.5) | 0 (0) | 1 | 1 (1.4) | 3 (6.5) | 0.298 |
| Headache | 1 (1.6) | 1 (2.0) | 1 | 0 (0) | 2 (6.9) | 0.222 | 0 (0) | 0 (0) | - | 1 (1.4) | 0 (0) | 1 |
| Vertigo | 0 (0) | 1 (2.0) | 0.451 | 0 (0) | 2 (6.9) | 0.222 | 0 (0) | 0 (0) | - | 1 (1.4) | 0 (0) | 1 |
| Skin Allergy | 0 (0) | 2 (3.9) | 0.201 | 0 (0) | 0 (0) | - | 0 (0) | 0 (0) | - | 0 (0) | 0 (0) | - |
| Erythema (Redness) | 6 (9.7) | 1 (2.0) | 0.126 | 0 (0) | 3 (10.3) | 0.102 | 1 (1.3) | 0 (0) | 1 | 4 (5.6) | 3 (6.5) | 1 |
| Warmth | 2 (3.2) | 2 (3.9) | 1 | 1 (3.1) | 1 (3.4) | 1 | 1 (1.3) | 1 (3.8) | 0.432 | 2 (2.8) | 2 (4.3) | 0.642 |
| Itch | 0 (0) | 1 (2.0) | 0.451 | 1 (3.1) | 1 (3.4) | 1 | 2 (2.5) | 0 (0) | 1 | 1 (1.4) | 1 (2.2) | 1 |
| Swelling | 3 (4.8) | 5 (9.8) | 0.464 | 1 (3.1) | 3 (10.3) | 0.338 | 0 (0) | 1 (3.8) | 0.245 | 4 (5.6) | 4 (8.7) | 0.710 |
| Phlebitis | 2 (3.2) | 2 (3.9) | 1 | 1 (3.1) | 1 (3.4) | 1 | 0 (0) | 1 (3.8) | 0.245 | 3 (4.2) | 2 (4.3) | 1 |
| Discharge | 0 (0) | 2 (3.9) | 0.201 | 1 (3.1) | 1 (3.4) | 1 | 0 (0) | 0 (0) | - | 1 (1.4) | 2 (4.3) | 0.560 |
| Bleeding | 0 (0) | 2 (3.9) | 0.201 | 1 (3.1) | 1 (3.4) | 1 | 0 (0) | 0 (0) | - | 1 (1.4) | 1 (2.2) | 1 |
| Pain | 30 (48.4) | 31 (60.8) | 0.188 | 25 (78.1) | 20 (69.0) | 0.417 | 33 (41.3) | 14 (53.8) | 0.261 | 36 (50.0) | 25 (54.3) | 0.645 |
